# LATE REACTION, PERSISTENT REACTION AND DOUBTFUL ALLERGIC REACTION: THE PROBLEMS OF INTERPRETATION

**DOI:** 10.4103/0019-5154.48989

**Published:** 2009

**Authors:** Nilendu Sarma

**Affiliations:** *From the Department of Dermatology, Venereology and Leprosy, NRS Medical College, Kolkata-700014, West Bengal, India*

**Keywords:** *Doubtful reaction*, *late reaction*, *persistent reaction*

## Abstract

The standard method of patch test reading is to read the test site for any positive allergy at 48 hr and then again at 72/96 hr. A late reading on the seventh day is also advised to exclude the irritant reaction (IR) and to notice some delayed development of allergic reaction. However, multiple visits are often difficult for the patient; therefore, this late reading is sometimes omitted. Here a case of plantar hyperkeratosis, due to allergic contact dermatitis, is reported with some insight into interpretation of the patch test. The patient showed delayed patch test reaction to formaldehyde and colophony, which has never been reported before.

## Introduction

The result of patch test is noted at the time of removal of patch (at day 2 or 48 hours) and again on day 3/4. A reading on 7^th^ day is however always advisable due to various reasons. A late reading at 7^th^ day may be useful in distinguishing an irritant reaction (IR) from an allergic reaction. IR rarely persists till day 7 and usually show a decrescendo pattern reaction unlike allergic one that often persist even more than this period except very weak reactions. Weak or doubtful reaction are often regarded as IR. Interpretation becomes difficult in situation where doubtful reaction shows delayed onset and persist longer because delayed development of test site reaction usually signifies an allergic nature. Clinical assessment plays determining role in many such complex situations. Significance of reactions persisting long duration has remained unanswered.

Result of patch test of the present case of plantar hyperkeratosis raised many of these issues those require careful evaluation.

## Case Report

**Figure 1:**
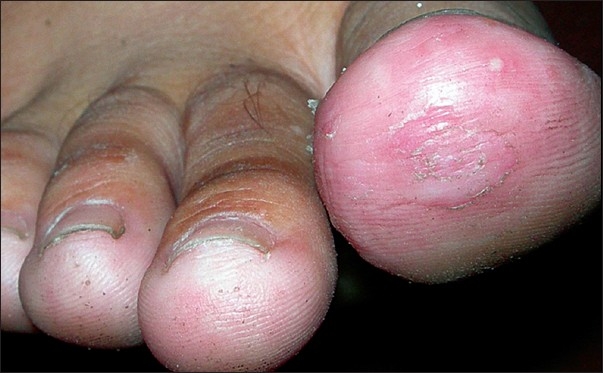
Hyperkeratosis involving toe tips

The present case is a Muslim female of 16 years, who presented with itchy hyperkeratosis over the tip of great toe for the last one year [Figure 1]. She had other forms of atopy, but not atopic dermatitis.

The patient was patch tested with Indian standard footwear series, with some additional allergens [Table 1].

**Table 1:** List of allergens tested in the patient

Allergens
Formaldehyde 1%
Mercaptobenzothiazole MBT 2%
Potassium dichromate 0.5%
Nickel sulfate 5%
Colophony
Epoxy resin 1%
Neomycin sulphate 20%
Monobenzyl ethar 20%
Thiurum mix 1%
Black rubber mix 0.6%
Glutaraldehyde 0.2%
Diocryl phthate 3%
Disperse orange 1%
Disperse blue 1%
Kathon CG 0.2%
4-Phenylenediamine (PPD) 1%
Chinoform 3%
p-tert Butylphenol formaldehyde resin 1%
Mercapto mix 2%

**Figure 2:**
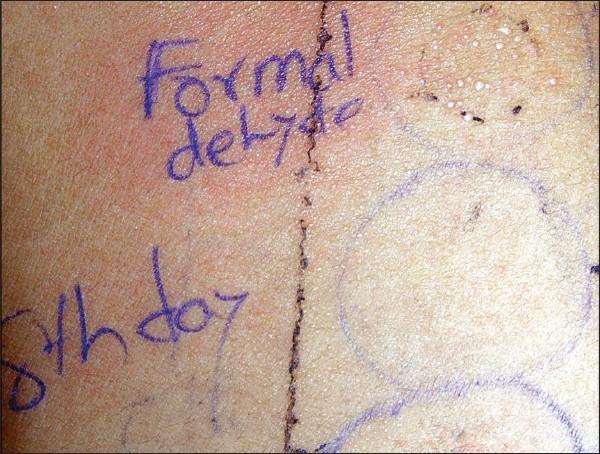
Vesicles at the test site of formaldehyde on the eighth day of reading

**Figure 3:**
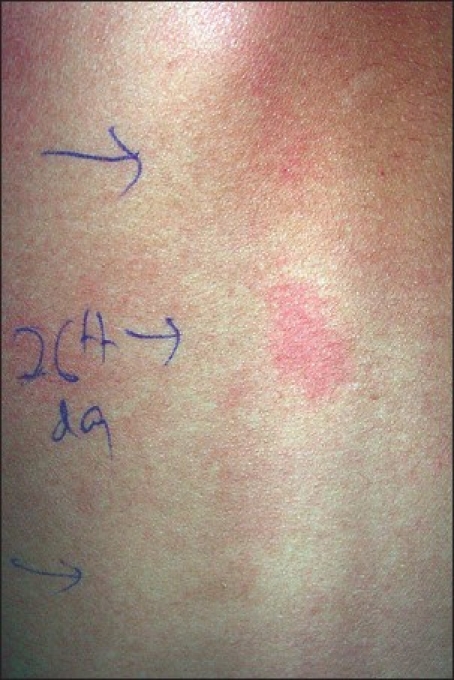
Erythema over chromate still persisting with mild erythema over formaldehyde on Day 26

The reading at 48 hr, 96 hr and on the sixth day revealed Grade I reaction against 0.5% potassium dichromate. On the eighth day, a few vesicles (Grade II), with faint erythema, developed at the test site of 1% formaldehyde and erythema on colophony. On the 15^th^ day, there were erythema and scales over formaldehyde and chromate, and erythema over colophony. On the 26^th^ day, formaldehyde and the chromate test site had persistent erythema [Figure 2, Figure 3].

The allergy to all three was found to be relevant, indicating leather, adhesive and plastics from footwear as the source.

## Discussion

The clinical presentation of the patient was very subtle. However, the result of patch testing produced interesting observation and provided scope for discussion on some important but rarely addressed core issues of interpretation.

There are some allergens those are sometimes reported to show a delay in the development of positive test against them. Neomycin, corticosteroids and gold can show late development of positive test. Therefore, they are sometimes called ‘slow’ allergens. Similarly, fragrance mix and Balsam of Peru etc. are called ‘early’ allergens.[1] It is said that late reaction can be found with any allergen with variable frequency and, thus, a late reading is highly appreciated in all patch test reading. However, we have not found any report of delayed development of positive test against formaldehyde and colophony. Formaldehyde developed higher grade of reaction with vesicles, starting later than chromate. Active sensitization is now regarded as very unlikely with the standard allergens, given the strength that is being used. Retesting has been planned but not done.

Although delayed irritation has also been reported, albeit only occasionally,[2] generally, reaction developing on or after the seventh day almost rules out an IR. Whether the doubtful reactions also, those develop late as seen in this case, would thus be called allergic reaction is not known.

Another important issue was the significance of doubtful reaction or mild erythema, without other signs. Interpretation of doubtful reaction remains difficult. Increasing the test dose and using a serial dilution is usually advocated. However, it is practically difficult to do so in most situations, except in some research centers.

Fisher followed some cases with weak reaction against phenylenediamine and dichromate, for even up to 20 years, but he did not found any higher grade when re-patch tested, nor did he find any clinical dermatitis attributable to those allergens. So he said that such weak reactions were of no clinical significance in many cases. However, he also stated that weak reaction or simply erythema, if it persists or increases after 48 hours, was probably allergic in nature.[3] Moreover, it is said that a relevant reaction, even if doubtful, can be taken as allergic reaction.[1] Till date, there is no consensus recommendation for correct assessment in these situations.

In the present case, the patient had prominent erythema on the eighth and the 15^th^ day. Retrospectively, it seemed that we probably missed a milder erythema at earlier reading. Or there could be true delayed development of reaction. It appeared that the doubtful reaction was allergic in nature, as it showed a crescendo pattern. This case highlighted the clinical significance of doubtful reaction.

This case highlighted the fact that we still require some consensus regarding some specific areas of patch test evaluation.

Long lasting reaction even persisting months after test has been reported in the past.[4] The exact reason for a persistent reaction is poorly understood.

The case also provided an opportunity for the first time, to see allergic reactions in patch test against formaldehyde and chromate, which persisted at least up to the 26^th^ day.
